# Patient perspectives and experiences of remote consultations in people receiving kidney care: A scoping review

**DOI:** 10.1111/jorc.12419

**Published:** 2022-03-25

**Authors:** Catriona Ewart, Jyoti Baharani, Martin Wilkie, Nicola Thomas

**Affiliations:** ^1^ Institute of Health and Social Care London South Bank University London UK; ^2^ University Hospitals Birmingham Birmingham UK; ^3^ Sheffield Teaching Hospitals NHS Trust Sheffield UK

**Keywords:** kidney disease, patient perspectives, remote consultation, scoping review

## Abstract

**Background:**

The coronavirus disease 2019 (COVID‐19) pandemic resulted in a rapid and sometimes chaotic change in how clinical care was delivered for people living with kidney disease, with increased reliance on digital technologies and the introduction of remote services.

**Objectives:**

To conduct a scoping review of studies about patients' experiences and perspectives in receipt of remote consultations for kidney care.

**Design:**

Using Arksey and O'Malley's framework, three databases were searched on EBSCO (CINAHL, MEDLINE and Psych INFO). The search included studies published in English from August 2010 to August 2021.

**Results:**

Eight studies met the scoping review criteria (two cross‐sectional, two mixed‐method and four qualitative). Five themes were identified: overall satisfaction with remote services, benefits to patients (convenience, involvement in care and patient safety), barriers to remote consultations (technical difficulties, digital literacy and loss of interpersonal communication), patient concerns (need for physical examination, privacy and confidentiality) and prerequisites for successful remote care (existing patient–practitioner relationship, stable illness phase and access to technology).

**Conclusion:**

Remote consultations confer multiple advantages to patients; therefore, remote consultations should be offered as an option to patients living with kidney disease beyond the COVID‐19 pandemic. However, there are several barriers to remote consultation that need to be addressed and understood before implementing remote care long‐term. Future research should examine the impact of remote consultations on people living with kidney disease from under‐served groups to identify barriers and ensure their suitability and accessibility to the wider population for a more patient‐centred approach to kidney care.

## INTRODUCTION

The coronavirus disease 2019 (COVID‐19) pandemic resulted in a rapid and sometimes chaotic change in how clinical care was delivered to people living with kidney disease, with the introduction of digital technologies and increased reliance on telehealth. Telehealth or remote care is a clinical practice, usually conducted by video call or telephone, to provide a clinical consultation (Sikka et al., 2019). Before COVID‐19, telehealth was initially implemented to maximise health‐care access to people living in rural and remote areas (Bashshur & Shannon, 2009; Fisk et al., 2020; Rohatgi et al., 2017). However, over the past decade, new technologies have widened the scope of telehealth practices, making these tools more accessible and effective in caring for patients regardless of geographic proximity (Kvedar et al., 2014).

Given the current coronavirus pandemic, remote consultations are being widely adopted so that people living with kidney disease can access care while maintaining social distancing without risking exposure to and spread COVID‐19. These changes are often heralded as being responsive, innovative, and person‐centred. However, there is a growing concern that such changes may adversely affect existing health inequalities in under‐served communities (Bonner et al., 2018; Stauss et al., 2021; Walker et al., 2019). Examples of under‐served groups include individuals with poor health literacy, who are not fluent in English, have a learning disability or cognitive or mental health issues (Stauss et al., 2021; Walker et al., 2019).

A systematic review of patients' experiences of real‐time online consultations at patients' homes for people living with chronic diseases (Almathami et al., 2020) (*n* = 45) reported benefits, including the reduced burden of travel and increased convenience. However, there were concerns about losing interpersonal contact and technical difficulties. None of the studies in this review included patients with kidney disease, and none included participants from under‐served groups. However, under‐served groups have specific characteristics such as high health‐care burden and significant differences in how they respond to or engage with health‐care interventions. Therefore, it is essential to better understand experiences of people living with kidney disease in receipt of remote care.

Currently, only two systematic review studies explore the factors influencing patients experience of remote consultations in kidney care (Blinkhorn, 2012; Lunney et al., 2018). However, these reviews focus primarily on the uptake of remote consultations to improve clinical health outcomes for those living with kidney disease. Despite the existing, albeit limited, evidence demonstrating the feasibility of remote consultations maintaining and even improving clinical health outcomes for patients living with kidney disease, there is little evidence to understand how patients experience and perceive remote consultations. Indeed, narrative reviews have discussed the potential applications of remote consultations for people living with kidney disease (Stauss et al., 2021). However, the impact of remote consultations and experiences of people living with kidney disease has not been systematically reviewed. Given this gap in the literature, a scoping review of the literature was undertaken to examine the available evidence. This scoping review aimed to explore the experiences and perspectives of people living with kidney disease toward remote consultations. Additionally, this scoping review aimed to map the range of literature on the topic to identify gaps that may inform future health‐care practices and research.

## METHODS

The current scoping review aimed to explore the experiences and perspectives of people living with kidney disease toward remote consultations in kidney care. The scoping review followed the five‐stage methodological framework developed by Arksey and O'Malley (2005) and included (1) identifying the research question; (2) identifying the studies relevant to the research question; (3) study selection; (4) charting the data; (5) collating, summarising, and reporting of the result. This framework allowed the inclusion of various study designs and broader topics, such as telephone and video consultations, to be thoroughly investigated.

### Search strategy

The inclusion of studies was limited to those about synchronous (i.e., video and telephone) remote consultations. Table 1 shows the inclusion and exclusion criteria.

**Table 1:** Inclusion and exclusion criteria

Selection criteria	Inclusion criteria	Exclusion criteria
Language	English language	Not English
Dates	Publications from 2010 to 2021	Publications from before 2010
Study type	Quantitative research	Conference abstracts, reports, and case studies, news articles and editorials, unpublished primary studies that were ambiguous and vague about remote care
	Qualitative research	
	Systematic reviews	
	Mixed methods	
	Empirical research	
Topics	Kidney disease, end‐stage kidney disease, renal care, remote care, remote consultations, video consultations, telephone consultations, patient experiences, attitudes, perspectives, feelings and opinions	Remote monitoring, mobile health, digital intervention, web‐based health, virtual reality, clinical outcomes, hospitalisations, comorbidities, practitioner or staff perspectives

Three large digital databases were searched within EBSCO (CINAHL, MEDLINE, and PsychINFO) for articles about remote consultations. Additionally, the reference lists of selected studies were checked for further appropriate articles. The search was performed in August 2021. After identifying the digital libraries, specific key works were searched for the required data. The search strategy included a wide range of key works to increase the sensitivity and inclusiveness of the search (Table 2).

**Table 2:** Search strategy

Step	Searched limiters	Results
1	Renal care OR kidney care OR kidney disease OR Chronic Kidney disease OR CKD OR Nephrology OR Dialysis OR Haemodialyses OR end‐stage kidney disease OR ESKD or end‐stage renal disease OR ESRD or end stage kidney failure OR ESKF OR renal replacement therapies OR RRT OR peritoneal dialysis OR HD	719,221
2	Remote consultation OR remote care OR video consultation OR telephone consultation OR telehealth	69,926
3	Attitudes OR perspectives OR experiences OR perception OR opinions OR thoughts OR feelings OR beliefs	5,219,276
4	1 AND 2 AND 3	150
5	Limiters—English	146
6	Limiters—Geography (United Kingdom and Ireland, Europe, North Territories, Canada, Australia)	131
7	Limiters—Year (August 2010 to August 2021)	115
8	5 AND 6 AND 7 AND Journal article OR Review	112

### Study selection

**Figure 1:**
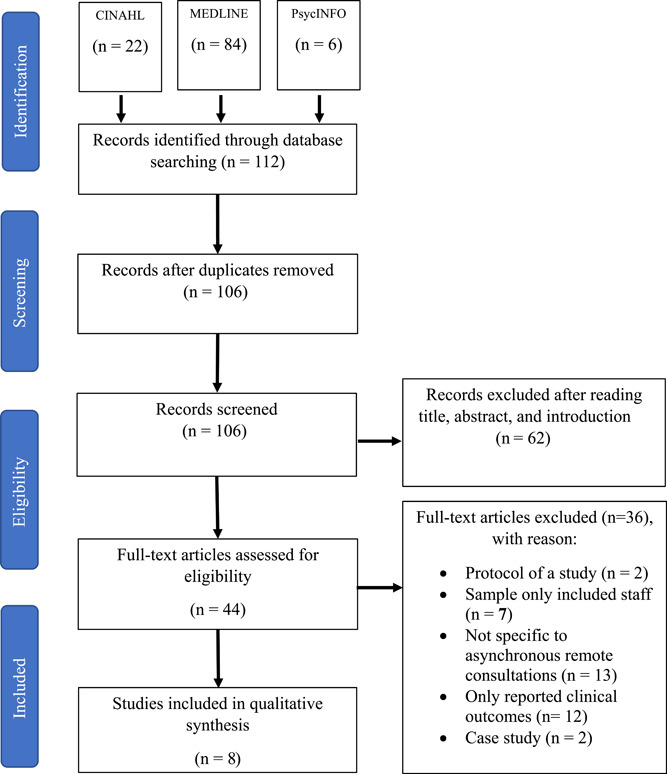
Search collection and screening process using PRISMA extension

The initial screening process identified 112 studies of which six were duplicates.  Figure 1 illustrates the search collection and screening process using preferred reporting items for systematic reviews and meta‐analyses (PRISMA) extension for the scoping reviews checklist (Tricco et al., 2018). After screening of titles, abstracts, and introductions, 44 were selected for full‐text review with only 8 meeting all the inclusion criteria.

### Data extraction

Study characteristics and information, such as publication year, study location, aim, design, sample size, and major findings, were documented and tabulated for the included studies (Table S3).

## RESULTS

A total of eight studies published between August 2010 and August 2021 were included in this scoping review. Two studies used a cross‐sectional design (Alshaer et al., 2020; Campbell et al., 2012), two used a mixed‐methods design (Lunney et al., 2021; Qiu et al., 2021), and four used a qualitative design (Huuskes et al., 2021, Lunney et al., 2020; Trace et al., 2020; Varsi et al., 2021). The studies were conducted in the United Kingdom, Canada, Australia, and Norway. Five common themes identified across the studies were (1) overall satisfaction with remote consultations, (2) benefits to patients, (3) barriers to remote consultations, (4) patient concerns, and (5) prerequisites for successful remote consultation.

### Overall satisfaction with remote consultations

Participants overall satisfaction with remote consultations was reported in seven of the studies included in this review. Across three studies, participants reported high satisfaction with video consultations, stating they wanted to continue with remote health‐care services, and would also recommend the service to others (Lunney et al., 2020, 2021; Varsi et al., 2021). Alshaer et al. (2020) found only 9% of participants rated the video appointment poorer than a face‐to‐face visit. While most patients (91%) did not express a compromise in the overall experience, rating it no different, better, or significantly better. Campbell et al. (2012) found that participants were satisfied with the session (76% strongly agreed), were able to present the same information as they would have in person (72% strongly agreed) and felt as confident about the doctor's assessment as they would with an in‐person assessment (77% strongly agreed). However, some patients reported that while there was nothing wrong with remote consultations, they simply preferred an in‐person visit with the doctor. Qiu et al. (2021) reported that 45% of adolescents preferred remote consultations and 55% preferred in‐person visits. Trace et al. (2020) reported an overall high satisfaction with video consultations and participants expressed that even where in‐person care is available, remote consultations should supplement in‐person care. Trace et al. (2020) and Qiu et al. (2021) both reported that participants preferred video consultations to telephone, especially if they had not met the practitioner in‐person before. In summary, remote consultations appear to offer an overall effective alternative to in‐person consultations, without compromising patients' health‐care experience.

### Benefits of remote consultations

#### Convenience

The benefits of remote consultations for patients were identified in seven studies included in this review. The most widely reported benefit was the convenience of remote consultations (Alshaer et al., 2020; Huuskes et al., 2021; Lunney et al., 2020; Qiu et al., 2021; Varsi et al., 2021). Participants reported that video consultations reduced the burden of appointment waiting time and time spent travelling. Moreover, the reduced burden of travel was further found to decrease travel time and costs associated with attending in‐person consultations, such as transportation, parking and childcare.

Varsi et al. (2021) further revealed participants perceived conducting consultations from the comfort of their own home as an added convenience. Patients reported feeling less stressed when attending a consultation from home rather than going to the hospital which came with feelings of anxiety and stress. Additionally, participants appreciated the flexibility of remote consultations, particularly not needing to cancel if they did not feel well enough to travel (Varsi et al., 2021). Alshaer and colleagues (2020) found that remote consultations were more convenient, with participants not having to take time off work/school or arrange childcare and reduced patients' reliance on caregivers. Huuskes and colleagues (2021) found that remote consultations interfered less with participants work as they no longer had to travel to or wait for clinic appointments. As such, participants reported feeling less guilt about disappointing their employer and felt they could better commit to their work.

#### Involvement in care

Two studies reported that participants perceived increased involvement with their healthcare as an added benefit of remote consultations. Trace and colleagues (2020) found that younger children enjoyed the novelty of using the computer and older children enjoyed the familiarity of screen‐based conversations. Furthermore, Trace et al. (2020) identified that the ability to screen‐share during video consultations increased understanding of medical information and family engagement. Parents noted that there is no time to process the information in a face‐to‐face clinic. However, screen‐sharing enabled understanding of their child's healthcare, giving parents some relief and encouragement to continue dietary interventions. Moreover, screen‐sharing allowed children to convene directly with the dietician rather than through a parent proxy by telephone. Huuskes et al. (2021) found that the increased responsibility for self‐management required for remote consultations such as taking blood pressure, weight, and temperature, participants reported a sense of empowerment and readiness in their health care.

#### Patient safety

Three studies reported the element of safety for patients as benefit to remote consultations. According to Huuskes and colleagues (2021), patients felt that remote consultations enabled them to stay home and avoid the risk of being exposed to infections in the clinic. Qiu et al. (2021) revealed that all participants felt that remote consultation was a safe way to communicate with their physicians. Alshaer et al. (2020) further revealed that in the context of COVID‐19 and considering the vulnerability of kidney transplant patients were among the most vulnerable group, many participants (77%) recognised that, due to their clinical condition, remote consultations offered a safer and easier mode of medical review.

### Barriers to remote consultation

#### Technical difficulties

Technical problems were identified as major barrier to remote consultations in six of the included studies, in that poor connection and video quality hindered the consultation experience. Qiu et al. (2021) revealed that almost all participants expressed dissatisfaction with the technical elements of remote consultations, including video and audio lags and internet disconnections during the consultation. For some participants, these pauses made it more difficult to remember things, such as instructions from the doctor. Both Huuskes et al. (2021) and Varsi et al. (2021) reported that participants found technical difficulties stressful, interrupting their communication and compromising their consultations experience. In some cases, participants reported having to continue the consultation by telephone when technical problems occurred (Varsi et al., 2021). Trace et al. (2020) found that 10/12 families reported technical difficulties in their consultation and often required telephone support from the dietician to resolve technical problems such as browser updates.

#### Digital literacy

Familiarity and experience using technology were identified as a barrier to conducting remote consultations in four of the studies. In this review, only three of the studies provided participants with technical training or information on conducting remote consultations. Varsi et al. (2021) reported that despite receiving information in advance about how to download and use the video consultation, some participants expressed concerns about feasibility of the process. Participants reported that the information may be too difficult to follow for people with less technical knowledge or experience and suggested that health‐care staff could offer more technical support for those who might need it. Similarly, Lunney et al. (2021) found that participants struggled using technology and some noted that if training had not been available, they might have had difficulty using the technology. Huuskes and colleagues (2021) revealed that some participants felt ill‐prepared for their first video appointment, including how to use the video call system and what health measurements they were required to provide, and wanted more detailed guidance on how to prepare for a remote consultation. Some participants felt that their doctors experienced a 'learning curve' and were not familiar with technology, resulting in a less effective consultation.

#### Loss of interpersonal communication

Six studies identified that participants perceived the loss of interpersonal communication between themselves and their practitioner as a barrier to remote consultations. In the study by Lunney et al. (2020), participants felt that nonverbal cues were missed in remote consultations due to the nature of communicating via video consultation. As such, patients felt the interaction was less personal and emotive indicators were lost through technology. According to Qiu et al. (2021), participants felt that the preset duration of the remote visits led to some patients feeling that video visits are rushed, with limited time to discuss all topics thoroughly. Similarly, in Lunney and colleagues' (2021) study, participants reported feeling pushed for time and that not all their questions would be answered. Lunney et al. (2020) reported that participants felt that their patient–practitioner relationships were impacted as appointments went much quicker and felt more routine, with less organic conversation and less opportunity to ask questions. Huuskes et al. (2021) revealed that participants faced challenges in hampering honest conversations when discussing sensitive topics such as mental health or personal concerns over video. In Qiu and colleagues' (2021) study, parents expressed, although not exclusively, that adolescents potentially take video visits less seriously, as the physician was not physically present. One caregiver noted that adolescents are held more accountable and actively engaged in their healthcare when meeting the doctor in person. Huuskes et al. (2021) reported that participants missed seeing, interacting and sharing experiences with other patients, which participants felt could help alleviate their concerns and normalise their kidney transplant experience.

### Patient concerns

#### Privacy and confidentiality

Four of the reviewed studies identified privacy and confidentiality of remote consultations as a concern to patients. Huuskes et al. (2021) reported that the confidential nature of information sharing during consultations meant that some participants were not comfortable sharing sensitive information in their own homes or workplace and preferred the privacy of the doctor's office when in‐person. Trace and colleagues' (2020) reported concerns raised about confidentiality, with participants noting that the practitioner was not always aware of who was present in the room at home. In the study by Varsi et al. (2021), concerns were raised regarding the leaking sensitive information or getting hacked by unauthorised persons and the need for strict hospital routine to be followed to reassure patients of their privacy and confidentiality. Varsi et al. (2021) further revealed that participants struggled to find a quiet and private place at work or home not to be overheard during video consultations. Huuskes et al. (2021) also reported the challenges of finding a conducive environment, as distractions at home such as 'dogs barks' or 'kids screaming' limited the ability of some participants to engage with their remote consultation fully.

#### Need for physical examination

Four of the studies reported the need for physical examination was perceived as concern for remote consultations. Qiu et al. (2021) reported that adolescents were more comfortable describing and showing their physician symptoms in‐person, rather than trying to explain symptoms via a screen. In the study by Lunney et al. (2020), participants reported that due to the nature of communicating via video, nonverbal cues were missed in remote consultations. As such, participants felt the limited ability for physical assessment meant they could be at risk of a missed diagnosis. Huuskes et al. (2021) further revealed that patients had difficulty conveying their symptoms via video or photographs. Moreover, participants felt that face‐to‐face appointments allowed clinicians to comprehensively observe their health to provide more candid advice. Similarly, Varsi et al. (2021) reported that several participants were concerned about the risk of overlooking essential signs and symptoms over video consultations. One participant reported an element of 'extra safety' when the doctor performs physical examinations rather than when patients self‐examine.

### Prerequisites for success

#### Existing patient–practitioner relationship

Three studies identified an existing patient–practitioner relationship as a prerequisite for successful remote consultations. Varsi et al. (2021) found that a mutual trusting patient–physician relationship established over many years was necessary. Participants in this study had an already established, long‐term trusting relationship with their nephrologist. As such participants felt that changing from in‐person to video did not significantly change their relationship as the consultations still followed a similar structure and covered the same topics. Lunney et al. (2020) noted that participants felt the nuances of conversation were lost and that the patient–practitioner relationship felt less personal. Huuskes et al. (2021) revealed that in the context of the pandemic, participants appreciated that everyone was adapting to technology and had to build relationships virtually. However, for participants who had an existing relationship with their practitioner, remote consultations meant that these relationships were not as sociable.

#### Access to digital technologies

Access to the internet and a personal device, such as a PC, tablet or smartphone, was a requirement to participate in all the studies included in this review. This suggests that access to technology and the internet is a primary prerequisite to successful remote consultations. Varsi et al. (2021) revealed that two participants had to purchase a new device due to a lack of compatibility with the virtual meeting platform required for the video consultation solution. Similarly, Lunney et al. (2021) found that not all patients had devices that worked with virtual meeting platforms. However, Huuskes and colleagues (2021) highlighted that engagement with remote consultations might be limited in communities where access to technology or internet connection is limited. Access to technology, the internet and digital literacy are crucial prerequisites for successful remote consultation.

## DISCUSSION

This review reveals insights into patients' experiences and perspectives associated with remote consultations as reported in the current literature. Five common themes were identified in this review, all of which need to be considered to facilitate engagement and participation in remote consultations for kidney care. The primary benefit reported by patients across all studies was the convenience of remote consultations with the reduced burden of travel, stating that this meant time saving and reduced travel costs. The main barriers described by patients across all studies were technical difficulties, digital literacy and loss of interpersonal communication. The primary concerns expressed by participants were privacy and confidentiality and the need for physical examinations. Finally, prerequisites for successful remote consultations included the patients being in a stable phase of their illness, with minimal complications, access to technology and having an existing, established and trusting relationship with their practitioner. The articles included in this review described significant technical difficulties, including poor quality images and sounds and connection issues. Despite challenges with technology, many studies reported that patients valued having remote consultations as an alternative to in‐person consultations at the hospital.

To our knowledge, this is the first scoping review aimed at identifying patients' experiences and perspectives remote consultations for kidney care at a multinational level. Our results suggest that patients' receiving kidney care tend to view remote consultations as generally acceptable given the potential to reduce the burden of in‐person visits for patients, increase patients' safety, and facilitate involvement in patients' own care. The perceived benefits of remote consultations highlighted in this review are consistent with the results of a previous review exploring patients experiences of remote consultations in primary care (Thiyagarajan et al., 2020) and people living with chronic diseases (Almathami et al., 2020). Almathami et al. (2020) also found that remote consultations were well received and accepted by patients, with the benefit of reduced time spent and money spent travelling being highly valued. In line with previous findings, it was found that remote consultations increase patients' involvement in their kidney care and engagement with medical information, but also patients described feeling safe (Leonardsen et al., 2020; Rahimpour et al., 2008; Rygh et al., 2012). Although previous studies have found withdrawal from remote care due to nonadherence and technical difficulties with remote care in some disease settings (Cruz et al., 2014; Gorst et al., 2014), remote consultations have been widely welcomed by high‐risk patients in the context of the COVID‐19 pandemic (Boehm et al., 2020).

Based on our results, patients described technology as a significant barrier to remote consultations. Our findings described the challenges and disruptions from technology that frequently occurred, consistent with many previous findings (Donaghy et al., 2019; Greenhalgh et al., 2018; Sturesson & Groth, 2018). Despite technical challenges, patients continued to persevere with technology and remained enthusiastic about remote consultations. Previous research has identified socioeconomic factors, education level and age are barriers to using technology for remote care (Kontos et al., 2014; Rosner et al., 2017). Therefore, it is possible that many of the participants within these studies were digitally literate and experienced technology users. Alternatively, previous research suggests that the perceived seriousness of patients' conditions can be an important factor influencing patients' willingness to use remote consultations, where lower use of remote consultations is associated with poorer health conditions (Mold & de Lusignan, 2015).

It was further found that patients experienced a loss of interpersonal interaction with remote consultations, which raised challenges in building a rapport with clinicians, communicating nonverbal cues and navigating sensitive conversations. The results from the current review underlined the perceived prerequisites for successful remote consultation. In line with previous research (Rubeis et al., 2018), we found that an established, trusting relationship between patient and physician was important for success. During the COVID‐19 pandemic, it was reported that physicians preferred to conduct remote consultations via video rather than over the phone, as this helped establish a rapport with people living with diabetes (Quinn et al., 2020). Moreover, this study acknowledges that physicians require remote consultation training was required for physicians.

Despite the manifold potential benefits of remote consultations for patients, the long‐term implementation of such practices demands careful consideration and evaluation of appropriateness and effectiveness for future kidney care.

### Strengths and limitations

The current scoping review had several strengths. The inclusion of quantitative and qualitative articles provided a broad approach to sourcing current literature. The studies included patients of different ages and across different countries, which offered a broader understanding of patients' experiences. Finally, this scoping review focused specifically on patients' experiences and perspectives toward remote consultations, providing a more patient‐centred understanding of the impacts of remote care.

This review is not without its limitations. The review only included studies published between 2010 and 2021, which may have contributed to the limited number of studies found. Publication and language bias should be acknowledged, especially as studies reflecting cultural differences in patients' experiences across countries may have been excluded. As this scoping review focused on remote consultations, these findings are limited to this modality of remote care and cannot be generalised to other modalities of remote care. There were concerns regarding the methodological quality of the reviewed studies, including small sample sizes and the homogeneity of the sampled populations. As such, the results of this review may not fully reflect the perspectives or experiences of those from different groups of people. Finally, the review did not include the experiences of the perspectives of practitioners working in kidney care. Although this review's goal was to provide a person‐centred understanding of remote consultations, the inclusion of renal staff's perspectives may have provided a more holistic understanding.

### Practical implications and future research

The findings of this scoping review have implications that the design of remote consultations require careful consideration of the factors facilitating or favouring remote practices. The findings potentially impact the future delivery of remote consultations, allowing health‐care providers to improve the delivery of remote consultations by implementing new practices and procedures, with a summary of recommendations outlined in Table 3.

**Table 3:** Summary of recommendations for future delivery of remote consultations

1. Continue the provision of remote consultations as an alternative to in‐person visits for people with kidney disease beyond the COVID‐19 pandemic.
2. Develop a hybrid model for long‐term follow‐up care to supplement remote consultations with face‐to‐face consultations.
3. Where possible, provide face‐to‐face consultations for first initial visit to build patient–practitioner rapport.
4. Provide training for practitioners on how to conduct a remote consultation and communicate health information virtually.
5. Incorporate training on remote consultation into undergraduate medical and nursing curricula, and in junior doctor education programmes.
6. Prepare and provide an information sheet/resource for patients to prepare for remote consultations.
7. Ensure technical support/training is available to both clinicians and patients.

Abbreviation: COVID‐19, coronavirus disease 2019.

*First*, we recommend that remote consultations be offered to patients living with kidney disease beyond the COVID‐19 pandemic. The manifold benefits of remote consultations on patients' lives, mainly the convivence, make remote care a viable option, with substantially reduced time spent travelling, time spent in waiting rooms, and the reduced costs. *Second*, we recommend that health‐care professionals strive to develop a Hybrid Model for long‐term follow‐up care by supplementing remote consultations with face‐to‐face consultations. However, patients' preference for remote or face‐to‐face consultation should always be considered, and face‐to‐face consultations should always be offered when the clinical condition changes or when a detailed explanation of a sensitive topic is required. *Third*, we recommend that, where possible, initial consultations should be conducted in‐person to help establish a relationship between patients and practitioners before starting remote consultations.

*Fourth*, we recommend that all renal practitioners receive training on how to conduct remote consultations and communicate health information virtually. Practitioners should be up to date on the most recent developments in confidentiality and privacy associated with video and telephone consultations. *Fifth*, we recommend that training on remote consultations is integrated into undergraduate medical and nursing curricula and junior doctor education programs. *Sixth*, we recommend that practitioners provide patients with resources (written information and video tutorials) to help patients prepare for remote consultations. This resource could provide guidance and reassurance on how to navigate technical challenges, create a conducive environment for consultations, and discuss sensitive topics. Additionally, this resource could include a 'checklist' for their consultation, such as what information to bring (e.g., blood pressure or weight measures), find a comfortable and appropriate space, and a list of questions to ask the practitioner. *Finally*, ensure technical support/training is available to clinicians and patients during consultations to reduce stress and facilitate successful consultations.

Health‐care providers can use the findings of this review and the recommendations outlined above as a guide to emphasise the benefits and minimise or eliminate the barriers of remote consultations and improve the future delivery of remote kidney care consultations. However, implementing remote care will require addressing barriers related to accessing digital technology for patients and improving digital literacy to make remote care more widely accessible to people living with kidney disease.

There is still a lack of robust evidence examining the barriers to remote care (Stevenson et al., 2019), particularly surrounding digital inequalities (Chesser et al., 2016). Although virtual care is a valuable tool to connect patients with health services, little is understood about the gaps in digital literacy and access within under‐served populations. Most evidence regarding the use of remote consultations has been focused on the general population worldwide (Rohatgi et al., 2017).

Future research should evaluate the impact of remote kidney care services on people from under‐served groups living with kidney disease. Evidence suggests that as the availability of digital health information has increased, other aspects of the digital divide have emerged, including computer literacy, health literacy and mismatch between desired and available digital health services (Chang et al., 2004). Remote care is an essential tool that could be leveraged to provide equitable access to kidney care worldwide. Patient involvement in development, implementation and utilisation of such solutions should be considered an integral part in health‐care initiatives. Therefore, we suggest that remote care interventions for managing kidney disease should be codesigned with patients living with kidney disease from under‐served groups to identify specific barriers and address individual needs for a more patient‐centred approach to care.

## CONCLUSION

The findings of this scoping review underline the current understanding of patients' experiences and perspectives of remote consultations for kidney care. Remote consultations appear to be generally accepted by patients and can offer great benefits to patients in terms of convenience, safety and engagement in healthcare. There are several barriers to remote consultations and valid concerns raised by patients which need to be addressed. This review provides important recommendations for the future practice of remote consultations. Future research should investigate the impact of remote consultations on patients' living with kidney disease from under‐served groups to better understand the unique barriers to individuals and created a more person‐centred approach to kidney care.

## AUTHOR CONTRIBUTIONS

*Project leader, conceived review, participated in theme development, read and approved the final manuscript*: Nicola Thomas. *Project assistant; searched the literature, developed the themes, drafted the manuscript, read and approved the final manuscript*: Catriona Ewart. *Helped to draft manuscript and approved the final manuscript*: Jyoti Baharani and Martin Wilkie.

## CONFLICTS OF INTEREST

The authors declare no conflicts of interest.

## Supporting information

Supporting information.
