# Supplementary material for: Patient perspectives and experiences of remote consultations in people receiving kidney care: A scoping review
Source: J Ren Care. 2022 Mar 25;48(3):143–53. doi: 10.1111/jorc.12419 (PMC9545432; doi:10.1111/jorc.12419)
Supplement: Supplementary file 1 — Supporting information. [file JORC-48-143-s001.docx]

TABLE 3: (Supplementary information). Summary of Studies Included in the Scoping Review

| **Lead Author/**  **Year** | **Country** | **Aim** | **Design** | **Sample** | **Provider** | **Setting** | **Key Findings** |
| --- | --- | --- | --- | --- | --- | --- | --- |
| **Alshaer, 2020** | United Kingdom | To understand renal transplant patients’ experiences of virtual clinics during the COVID-19 pandemic | Cross-sectional (QUANT)  Self-report survey (Check-all-that-apply response and open text box)  Descriptive statistics | Renal transplant patients (*n* = 44, *Mdn* age = 50-64, male = 55%, ethnicity = 55% white) | Nephrology consultants (*n* =5), Transplant specialised nurse (*n* = 1) | Home | - Patients reported video consultations were an acceptable alternative to in-person consultations with where patients have pre-consultation and access to blood pressure readings - Video consultations could replace traditional consultations, particularly for long-term transplant clinics where patients do not necessarily need to be examined - Financial and travel-related stress relief was patients most highly reported positive feedback |
| **Campbell, 2012** | Canada | To determine whether telemedicine was a feasible way to provide health care and support to rural communities and to assess the level of satisfaction among patients and health-care providers with this care modality. | Cross-sectional  (QUANT)  Self-report survey (Likert-scale response and opened-ended questions)  Descriptive statistics | Patients with chronic kidney disease (not on dialysis) (*n* = 92, male = 63%, *Mdn* age = 65+) | Nephrologists (*n* = 8), Specialist nurses (*n* =7) | Remote site | •        Participant’s responses were overall positive and wanted telemedicine to continue  •        Some patients reported they would discontinue follow-up care if they had to travel to Ottawa.   - Telemedicine was found to be a feasible option for follow-up care of remote chronic kidney disease patients |
| **Huuskes, 2021** | Australia | To describe the perspectives of kidney transplant recipients on the benefits, challenges, and risks of telehealth | Cross-sectional (QUAL)  Focus Groups (*n* = 5-10)  Thematic analysis | Renal transplant recipients (*n* = 34, *Mdn* age = 31-50, male = 47%, ethnicity = 79% white) | Nephrologist | Home | - Five themes were identified: minimizing burden, attending to individual context, protecting personal connection and trust, empowerment, and readiness; navigating technical challenges - Overall, telehealth was found to be convenient and reduce time, financial, and overall treatment burden - Following the pandemic, telehealth should be provided by a trusted nephrologist and accompanied with resources to help patients prepare for appointments |
| **Lunney, 2020** | Canada | To evaluate perceptions of patients and providers to inform the future design of a rural eVisit program specific to maintenance haemodialysis care. | Cross-sectional  (QUAL)  Semi-structured Interviews  Thematic Analysis | Patients on haemodialysis (*n* = 11, *Mdn* age = 66, male = 73%)  (*n* = 11, 73% = male, *Mdn* age = 66) | Nephrologist (*n* = 5), renal nurses (*n* = 8) | Remote site | •        Overall, eVisits may be useful modes of consultations in rural haemodialysis settings   - Patients reported that eVisits would save patients time and money and allow for more accessible and convenient care - Most patients reported that technical challenges, risk of missed diagnosis, and negative impact on patient-provider relationships were potential disadvantages |
| **Lunney, 2021** | Canada | To (1) design and test a virtual (video) kidney   clinic   model   for   routine   visits   between   patients on haemodialysis and their nephrologist and (2) identify the barriers and facilitators of video visits that may guide future implementation. | Cross-sectional (Mixed methods)  Self-report survey  Descriptive statistics  Individual Semi- Structured Interviews  Thematic analysis | Haemodialysis patients (*n* = 8, male = 62%, *Mdn* age = 58) | Nephrologists (*n* = 7) | Remote site | - Overall patients reported satisfaction with video consultation service, willing to use it again, and would recommend it to others - Three themes were identified with respect to factors influencing visit success: IT infrastructure, administration, and process |
| **Qiu, 2021** | Canada | To explore the attitudes of adolescent chronic paediatric nephrology patients and their caregivers towards telemedicine visits in comparison to in-person visits to our tertiary centre. | Cross-sectional (Mixed methods)  Self-report survey  Descriptive statistics  Individual structured telephone interviews  Thematic analysis | Paediatric nephrology patient dyads (*n* = 11, male = 45%, *M* age = 14.4 (± 2.5) | Paediatric Nephrologist | Remote site | •     Experiences with telemedicine were characterized by *consultation-specific factors* and *contextual factors.* Contextual factors such as travel, and cost saving were valued by adolescents and caregivers. Consultation specific factors such as showing the doctor physical symptoms during in-person consultation were particularly valued by adolescents   - Overall, preference of visit modality was influenced by consultation nature. For less complex needs and regular check-ups, participants felt telemedicine was comparable to in-person visits |
| **Trace, 2020** | United Kingdom | To investigate whether virtual dietetic consultations are acceptable to parents and children | Cross-sectional (QUAL)  Semi-structured interviews  Inductive framework analysis | Families (*n* = 12)  Children (*n* = 5, male = 80%, *M* age = 9.4)  Parents (*n* = 13) | Renal Paediatric Dietitians | Home | •         Six themes were identified including *logistics, understanding information, establishing trust, willingness to change, family engagement,* and *preferences.*   - Overall, satisfaction with the video consultations was high, with no data security fears and only minor privacy concerns - Screen-sharing software was found to enhance parents understanding, generate greater discussion and engagement - Parents requested that video consultations continue to supplement care, reporting improved access to specialist advice and efficiency - All children preferred video consultations |
| **Varsi, 2021** | Norway | To investigate, from the perspective of patients and health care providers, the perceived benefits and challenges of using video consultations in outpatient renal transplant recipient follow-up. | Cross-sectional (QUAL)  Semi-structured interviews  Thematic analysis | Renal transplant patients (n = 15, *Mdn* age = 53, male = 53%) | Nephrologist (n = 1), health support personnel (*n* = 2) | Home | •       Despite the technical deficiencies of the video consultation platform, participants appreciated the ability to alternate between in-person and video consultations   - Main benefits identified was reduced travel time and time saving, less focus on being chronically ill, possible economic benefits for patients and society - Facilitators for successful video consultations included stable illness phase, existing and trusting relationships with nephrologist - Potential harms and challenged reported included security, confidentiality, interruptions, and the need for physical examinations. |
